# The effect of combined cryoprotectants on the cryotolerance of boar sperm

**DOI:** 10.5713/ab.24.0915

**Published:** 2025-04-28

**Authors:** Shuangyi Deng, Liwei Yang, Li Gao, Chengcheng Ning, Shiyin Wang, Wei Zhang

**Affiliations:** 1 Key Laboratory of Livestock and Poultry Healthy Breeding Technology in Northwest China, Xinjiang Agricultural Vocational and Technical University, Changji, China

**Keywords:** Boar Sperm, Cryoprotectants, Cryotolerance, Frozen Dilute, Sperm Quality

## Abstract

**Objective:**

The frozen semen has the significant advantages of long-term storage and long-range transportation. However, due to the low cryotolerance of boar sperm, the global utilization of frozen boar semen in artificial insemination was less than 1% until the year 2000.

**Methods:**

In this study, the effects of five cryoprotectants at different concentrations on the cryotolerance of boar semen were evaluated when they were added separately, and the optimal concentrations for each cryoprotectant were determined, then their combined additive effects were further assessed.

**Results:**

At a glycerol (GLY) concentration of 5%, the quality of frozen-thawed sperm reached its maximum value, which was significantly higher than the 4% GLY group (p<0.05) and 0% GLY group (p<0.01). The straight-line velocity (VSL), curvilinear velocity (VCL), average path velocity (VAP), sperm plasma membrane integrity (SPMI), sperm acrosome integrity (SAI) and sperm mitochondrial activity (SMA) of the frozen-thawed sperm in treated egg yolk group exhibited significant improvements compared to the untreated egg yolk group (p<0.05). The total motility (TM), progressive motility (PM), SPMI, SAI, and SMA of 2% Equex STM paste group were significantly higher than the rest groups (p<0.05). The TM, PM, VSL, VCL, and VAP of frozen-thawed sperm in the 250 nM and 300 nM Mitoquinone mesylate groups showed significant improvements compared to the other groups (p<0.05), and the reactive oxygen species levels in sperm cells were also significantly lower (p<0.05). The quality of frozen-thawed boar sperm in 0.6 mM L-ergothioneine group reached its peak value and was significantly higher than the rest groups (p<0.05). When these five cryoprotectants were used in combination, the quality of frozen-thawed boar sperm exhibited a significant improvement compared to when they were used individually (p<0.05). Utilizing the frozen-thawed boar semen to inseminate estrus sows, the reproductive performance of the sows did not differ significantly from the sows inseminated with fresh semen (p>0.05).

**Conclusion:**

The optimized boar semen cryopreservation system can substantially enhance the quality of frozen-thawed boar sperm, making it suitable for artificial insemination in pig farm.

## INTRODUCTION

By employing proper procedures, animal sperm can be frozen and stored in liquid nitrogen (−196°C), significantly extending its viability outside the body. Since 1949, when glycerol (GLY) was identified as an effective cryoprotectant and a relatively stable sperm freezing protocol was established [1], sperm cryopreservation has been widely utilized in various fields, including the preservation of animal genetic resources, conservation of endangered species, cross-regional breeding of livestock, and the establishment of human sperm bank [2]. Particularly in livestock breeding, in conjunction with other technologies such as synchronized estrus, artificial insemination, embryo transfer, and sex control technologies, the use of frozen sperm has greatly improved the efficiency of livestock breeding [3].

The delicate structure of animal sperm limits its ability to adapt and resist various adverse conditions [4]. During cryopreservation, sperm is exposed to multiple detrimental factors, including rapid temperature decline, changes in osmotic pressure, mechanical damage from ice crystal formation, and oxidative stress. These factors can lead to structural impairments, functional disorders, and decreased motility [5]. Additionally, the cryotolerance of animal sperm is influenced by various factors, leading to the post-thawed sperm exhibit distinct levels of quality [4]. Boar sperm exhibits high sensitivity to the stress factors during cryopreservation. The motility, viability, conception rate (CR) and litter size of post-thawed boar sperm were relatively low [6]. This is the main reason for the global adoption of frozen boar sperm for artificial insemination remained below 1% until 2000 [7]. Currently, porcine artificial insemination primarily employs liquid semen preserved at a temperature of 17°C. Compared to cryopreservation, liquid storage of boar semen at 17°C offers several advantages including lower production costs, reduced damage to sperm, simplified operational procedures, and higher CRs. However, due to the limited storage duration of 3 to 7 days, this method is not suitable for long-term preservation of boar semen or for facilitating cross-border and inter-regional exchange of high-quality breeding pig semen [8].

China is the largest pork producer and consumer worldwide, with pork accounting for over 60% of the country’s total meat consumption. Consequently, the pig industry holds a significant place in Chinese economic and social development [9]. Since the first entry of African swine fever into China in 2018, pig breeding companies have implemented stringent closed-herd management practices to prevent the virus from entering their facilities. While these measures have effectively safeguarded biosecurity, they have also impeded the continuous genetic improvement of pigs by disrupting normal gene exchange between breeding farms. Due to the frozen sperm can be stored for a very long time, so there is enough time to detect whether the boar and frozen semen contain pathogens after the sperm were frozen. This enables pig farms to acquire a sufficient supply of pathogen-free frozen semen in a single purchase, rather than introducing live boars or fresh semen (FS) from other farms continually. By doing so, farms can maintain biosecurity while engaging in gene exchange and interregional collaborative breeding. Therefore, developing a stable and efficient boar sperm cryopreservation technology, producing high-quality frozen boar sperm, and utilizing in pig farm are crucial for Chinese pig industry.

During the semen freezing and thawing process, the plasma membrane of sperm is particularly susceptible to damage due to direct exposure to various stressors [10], such as the mechanical damage from ice crystals, cold stress caused by rapid temperature drops [11], and oxidative stress generated by reactive oxygen species (ROS) [12]. These damage to the sperm membrane structure can further compromise other cellular structures and functions. Therefore, reagents and methods that can protect the integrity of the sperm membrane would enhance their cryotolerance and post-thaw viability [13].

The aim of the present study was to investigate the effects of the cryoprotectants on improving the cryotolerance of boar sperm and to establish a highly efficient boar sperm cryopreservation system for use in pig farms.

## MATERIALS AND METHODS

### Animal management and sperm collection

Five Landrace boars, aged 2 to 3 years, were selected for semen sample collection. These boars were individually housed at an Artificial Insemination Centre (Tecon, Xinjiang, China), which could automatically regulate the temperature, humidity, and air quality within the household environment, and were fed a standard commercial diet tailored on their nutritional needs.

Semen samples rich in sperm were collected using the gloved-hand method. A total number of 40 ejaculates (8 ejaculates per boar) were collected from the five boars twice a week. The semen quality was assessed with a computer-assisted sperm analyzer (CASA; SCA-Sperm Class Analyser; Microptic S.L., Barcelona, Spain). Only semen samples with a total motility (TM) exceeding 90% were pooled to reduce individual differences. Subsequently, the pooled semen samples were diluted at a ratio of 1:4 (v:v) in Beltsville Thawing Solution (BTS; Minitube, Tiefenbach, Germany). These samples were then transported to the laboratory within two hours using a 17°C incubator.

### Experimental design

In this study, we employed a two-step method to dilute the boar semen prior to freezing. In the first step, the semen samples were centrifuged at 17°C (800×g for 15 min). Following the removal of the supernatant, the sperm pellets were re-suspended using a Glucose-Sodium citrate-Tris based Frozen Diluent I, which has been optimized in our laboratory, to achieve a final concentration of 2×10^9^ sperm/mL. Subsequently, the temperature of the diluted semen was gradually reduced from 17°C to 4°C over a 3 hours period.

In the second step, the sperm were diluted once more using Frozen Diluent II to a final concentration of 1×10^9^ sperm/mL at 4°C. The Frozen Diluent II was prepared by incorporating appropriate amounts of cryoprotectants into Frozen Diluent I. In this study, we selected five types of cryoprotectants: GLY (Solarbio, Beijing, China), Mitoquinone mesylate (MitoQ; Solarbio), L-ergothioneine (EGT; Solarbio), Equex STM Paste (ESP; Minitube), and fresh egg yolk (EY). It is crucial to ensure that both the diluents and the semen are at the same temperature during both dilution steps. To evaluate the effects of these cryoprotectants on the cryotolerance of boar sperm and to determine their optimal concentration, we conducted four experiments.

In Experiment I, the optimal concentration of GLY was determined, and its addition to Frozen Diluent II set at 0%, 4%, 5%, 6%, and 7% (v/v), respectively.

In Experiment II, the effect of high-pressure homogenized EY on cryotolerance of boar sperm was investigated. Fresh EY was preprocessed using a Microfluidization High Pressure Homogenizer (NanoGenizer; Genizer, Irvine, CA, USA) at a pressure setting of 10,000 psi. The treated EY (TEY) was then incorporated into Frozen Diluent I at a concentration of 20% (v/v) for the experimental group, while the untreated fresh EY (UTEY) served as the control group at the same concentration. GLY was included at the optimal concentration determined in Experiment I.

In Experiment III, the optimal concentrations of rest three cryoprotectants were determined when they were individually added to Frozen Diluent II. The concentrations of GLY and EY were based on the data from Experiments I and II. Specifically, their final concentrations in Frozen Diluent II were as follows: MitoQ (0, 150, 200, 250 and 300 nM), EGT (0, 0.3, 0.6, 0.9 and 1.2 mM), and ESP (0%, 1%, 2%, 3% and 4% [v/v]), respectively.

In Experiment IV, to evaluate the effect of combined cryoprotectants on boar sperm cryotolerance, these five cryoprotectants were added in combination at their optimal concentrations as determined in Experiment I, II, and III.

### Freezing and thawing of diluted semen

The boar semen, after being diluted with Frozen Diluent II, was further equilibrated for 1 h at 4°C. Subsequently, the semen was loaded into 0.5 mL straws and sealed using a semen filling and sealing machine (GREEN AURIS, China), with each straw containing 5×10^8^ sperm. The straws were placed on a specialized shelf and frozen in a programmable freezer (GREEN AURIS) using liquid nitrogen as the coolant. The freezing process comprised two stages: initially, the temperature was reduced from 4°C to 0°C at a rate of 0.06°C per second, followed by a decrease from 0°C to −140°C at a rate of 0.8°C per second. Upon completion of the freezing process, the straws were immersed in liquid nitrogen for long-term storage.

The straw containing the frozen semen was placed in a 50°C water bath for 16 seconds to facilitate rapid thawing. During this process, the straw must be continuously agitated to ensure consistent contact with the 50°C water, which is crucial for achieving optimal post-thaw viability. Subsequently, the straw was transferred to a 37°C water bath for temporary storage. The post-thawed semen was then immediately diluted 1:4 (v:v) with BTS and incubated at 37°C to assess its quality.

### Assessment of sperm motility and biokinetic parameters

A 5 μL aliquot of post-thawed boar semen, diluted 1:4 (v:v), was loaded into a MICRON sperm counting chamber (10 μm deep, GREEN AURIS, China) which was pre-warmed to 37°C. Sperm motility and biokinetic parameters were assessed using the CASA system. In present study, five parameters were measured: TM (defined as sperm with an average path velocity [VAP] of ≥20 μm/s), the progressive motility (PM; defined as sperm with an average straight-line velocity [VSL] of ≥40 μm/s), VSL (μm/s), curvilinear velocity (VCL, μm/s), and VAP (μm/s). Each sample was analyzed at 37°C and 100× magnification, with nine fields containing a minimum of 500 sperm being examined.

### Evaluation of sperm plasma membrane integrity

The hypo-osmotic swelling test (HOST) was employed to assess the plasma membrane integrity of post-thawed boar sperm. A 1 mL hypo-osmotic solution (comprising 0.506 g sodium citrate, 0.982 g fructose, and 100 mL distilled water) was prepared in an Eppendorf tube and incubated in a 37°C water bath for 5 min. Subsequently, 0.1 mL of post-thawed boar sperm was added and gently mixed. The tube was further incubated for an additional 30 min, after which 10 μL of the sample was placed on a microscopic slide with a cover slip. Coiled sperm (HOST positive) were counted under a phase-contrast microscope (CX41; Olympus, Tokyo, Japan) at 400× magnification. A total of 200 sperm per sample were counted, and each sample was analyzed in triplicate.

### Evaluation of sperm acrosome integrity

The integrity of the sperm acrosome was evaluated using the Fluorescein Isothiocyanate labeled Peanut Agglutinin (FITC-PNA) staining method. Following to the manufacturer’s protocol (Sigma-Aldrich, Taufkirchen, Germany), 0.5 mL of post-thawed semen was mixed with 1 mL of pre-cooled acetone (−20°C) and fixed at 4°C for 5 min, followed by centrifugation at 500 g for 6 min. The supernatant was discarded, and the sperm pellet was washed twice with phosphate buffered saline (PBS). To block non-specific binding sites, the sperm were treated with n-2-hydroxyethylpiperazine-n’-2-ethanesulfonic acid and bovine serum albumin solution for 30 minutes. The sperm were then incubated with FITC-PNA (0.01% in PBS) for an additional 30 minutes. After another centrifugation at 500×g for 6 min, the supernatant was discarded again, and the sperm pellet was washed twice with PBS and resuspended using 10 mL of PBS. Finally, a 20 μL aliquot of sperm suspension was placed on a slide and examined under a fluorescence microscope. Sperm exhibiting strong green fluorescence in the acrosomal region were classified as having intact acrosomes. A total of 200 sperm were counted per slide to determine the percentage of intact acrosomal sperm, and each sample was analyzed in triplicate.

### Evaluation of sperm mitochondrial activity

Mitochondrial activity was evaluated by quantifying changes in mitochondrial membrane potential using the JC-1 fluorescent probe. The experimental protocol adhered to the manufacturer’s instructions (Solarbio). 0.5 mL of post-thawed boar sperm was diluted in 1 mL of PBS and centrifuged at 500×g for 6 min. The supernatant was discarded, and the sperm were resuspended in 1 mL of PBS and mixed with 0.5 mL of JC-1 staining solution. The mixture was gently inverted several times and incubated at 37°C for 20 min. Following incubation, the sample was centrifuged at 500×g for 6 min at 4°C. The supernatant was removed, and the sperm pellet was resuspended in 1 mL of JC-1 staining buffer and subjected to another centrifugation under the same conditions to wash the sperm. This washing step was repeated once more. The sperm was then resuspended in 1 mL of JC-1 staining buffer. Finally, 20 μL of the sperm suspension was evenly spread on a slide and examined under a fluorescence microscope. Sperm exhibiting red fluorescence indicated normal mitochondrial membrane potential, whereas those emitting green fluorescence suggested a decreased in mitochondrial membrane potential. A total of 200 sperm per slide were analyzed, with each sample being replicated three times.

### Evaluation of reactive oxygen species level in sperm cell

A 0.5 mL volume of post-thawed boar sperm was added to 1 mL of PBS, followed by centrifugation at 500×g for 6 minutes. The supernatant was discarded, and the sperm was resuspended in 0.5 mL of PBS. Next, 50 μL of the diluted semen was mixed with 2 μL of a 10 μM 2’,7’-dichlorodihydrofluorescein diacetate (DCFH-DA) fluorescent probe (Sigma-Aldrich) and incubated in the dark at 37°C for 30 min. During this period, the sample was mixed every 3 to 5 minutes to ensure thorough contact between the probe and the cells. Following incubation, 500 μL of PBS was added, and the sample was centrifuged at 500×g for 6 min to wash it. This washing step was repeated three times to completely remove any unincorporated DCFH-DA. Finally, the sperm was resuspended in 400 μL of PBS, and the fluorescence signal was detected using a full-wavelength spectrophotometer (Multiskan Sky; Thermo Scientific, Waltham, MA, USA) at an excitation wavelength of 488 nm and an emission wavelength of 525 nm. Each sample was measured in triplicate.

### Artificial insemination and efficacy evaluation of frozen-thawed boar sperm

A total number of 10 ejaculates (2 ejaculates per boar) were collected from the five boars using the gloved-hand method twice a week. The optimized frozen diluent was utilized for the cryopreservation of boar semen with TM exceeding 90%, and only the frozen-thawed semen (FTS) with PM exceeding 60% was selected for subsequent artificial insemination experiments. A total of 100 Landrace sows in excellent health condition, aged 2 to 4 years and weaned from the same batch, were randomly divided into two groups: the control group and the experimental group, each consisting of 50 sows. All sows were housed within a same pen and further organized into smaller groups, each group comprising 5 sows. Each group of 5 sows was allocated to a 10-square-meter field, with a total of 20 fields designated for both the experimental and control groups. All sows were managed by a same breeder, who ensures uniform feeding protocols, and estrus detection was conducted twice daily, at 8:00 AM and 8:00 PM respectively. Sows exhibiting typical estrus signs were inseminated immediately upon detection. The control group received artificial insemination using FS, while the experimental group received FTS. Both groups underwent deep intrauterine insemination twice during estrus, with a 12-hours interval between injections, delivering a total of 2 billion motile sperm per injection. Following artificial insemination, the sows were monitored for two consecutive estrous cycles. The sows that did not exhibit estrus for two consecutive cycles were considered pregnant, and the CR was calculated for each group. After parturition, the number of inseminated sows, fertilized sows, parturient sows, live births, and total litter size were recorded. Subsequently, the CR, parturition rate (PR), average litter size (ALZ), and average number of live births (ALB) were calculated for each group. The CR of sows in each group was determined by calculating the proportion of sows that did not exhibit estrus over two consecutive estrus periods to artificially inseminated sows. The PR of each group sows was obtained by calculating the proportion of farrowing sows to artificially inseminated sows. The total number of piglets delivered by each group sows divided by the total number of farrowing sows was the ALZ for the group, and the ALB of each group sows was calculated by dividing the total number of live piglets by the total number of farrowing sows in this group.

### Statistical analysis

All statistical analyses were conducted using IBM SPSS 25.0 (SPSS, Chicago, IL, USA). The Shapiro-Wilk test was utilized to assess whether the data adhere to a normal distribution. The semen quality related data that exhibited a normal distribution were analyzed using one-way analysis of variance (ANOVA) followed by the Tukey Honestly Significant Difference test. The Chi-square test (χ^2^ test) was utilized to assess the statistical significance of differences in CR and PR, while the Student’s t-test was used to compare the differences in ALZ and ALB between the experimental group and the control group sows. A value of p<0.05 was considered to indicate statistical significance and a p<0.01 was considered to indicate highly significant difference. Results were presented as the mean±standard deviation, and each experimental group was replicated three times.

## RESULTS

### The effect of glycero on the quality of frozen-thawed boar sperm

Compared with the control group, the addition of GLY to the frozen diluent significantly improved the cryotolerance of boar sperm (p<0.01, Table 1). When the GLY concentration in the Frozen Diluent II was 5%, the TM, PM, VSL, VCL, and VAP of frozen-thawed boar sperm reached its maximum value, which was significantly higher than the 4% GLY group (p<0.05) and the control group with 0% GLY (p*<*0.01). There was no significant difference in TM among the 5%, 6%, and 7% GLY group (p>0.05), but the PM of the 5% GLY group was significantly higher than that of the 7% GLY group (p*<*0.05). Additionally, the VSL, VCL, and VAP values for the 5%, 6%, and 7% GLY groups were not significantly different (p>0.05), but they were significantly higher than those of the 4% GLY group (p<0.05) and the control group with 0% GLY (p<0.01).

**Figure 1:**
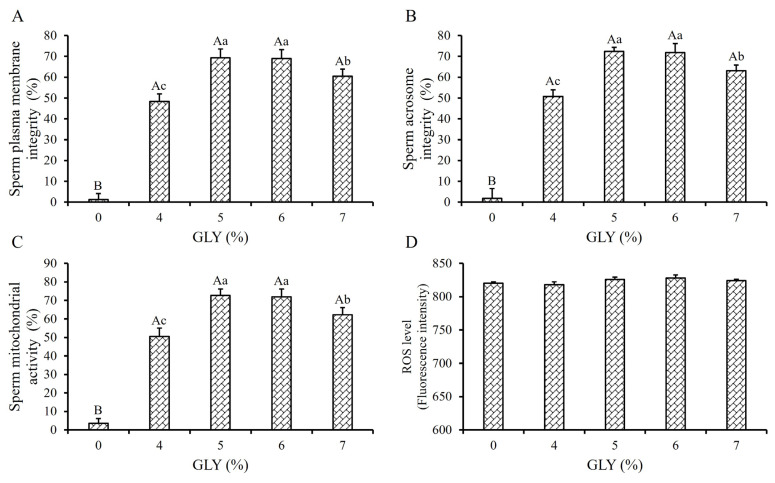
Effect of GLY on frozen-thawed boar sperm. Effect of different concentrations of GLY in Frozen Diluent II on the SPMI (A), SAI (B), SMA (C), and sperm ROS level (D) respectively. The same letter or no letter indicate p>0.05. ^a–c^ Different lowercase letters indicate p<0.05. ^A,B^ Different capital letters indicate p<0.01. GLY, glycerol; SPMI, sperm plasma membrane integrity; SAI, sperm acrosome integrity; SMA, sperm mitochondrial activity; ROS, reactive oxygen species.

The addition of 4%, 5%, 6%, and 7% GLY in the Frozen Diluent II significantly enhanced the sperm plasma membrane integrity (SPMI), sperm acrosome integrity (SAI), and sperm mitochondrial activity (SMA) of the frozen-thawed boar sperm (p<0.01, Figure 1), which may underlie the observed improvements in TM and PM of frozen-thawed sperm (Table 1). In addition, the SPMI, SAI and SMA of the 5% and 6% GLY groups were significantly higher compared to the 4% and 7% GLY groups (p<0.05, Figures 1A–1C). However, the addition of GLY did not affect the ROS levels in sperm cells (p>0.05, Figure 1D).

Therefore, a GLY concentration of 5% was selected for Frozen Diluent II in this study.

### The effect of high-pressure homogenized egg yolk on the quality of frozen-thawed boar sperm

**Figure 2:**
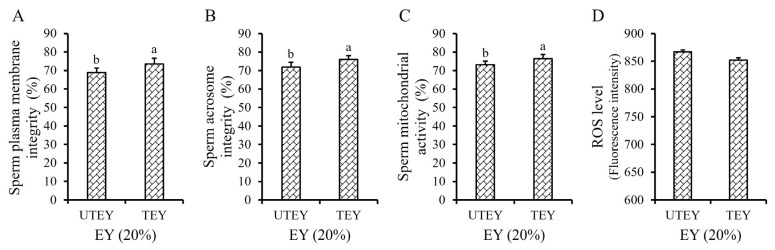
Effect of high-pressure homogeneous EY on frozen-thawed boar sperm. Effect of high-pressure homogeneous EY in frozen diluent on the SPMI (A), SAI (B), SMA (C), and the sperm ROS level (D) respectively. The same letter or no letter indicate p>0.05. ^a,b^ Different lowercase letters indicate p<0.05. UTEY, untreated egg yolk; TEY, treated egg yolk; EY, egg yolk.

When the GLY concentration in the Frozen Diluent II was 5%, the TM and PM of the frozen-thawed sperm in the TEY group were slightly higher than those in the UTEY group (p>0.05, Table 2). However, the VSL, VCL, VAP, SPMI, SAI and SMA of the frozen-thawed sperm in the TEY group exhibited significant improvements compared to the UTEY group (p<0.05, Table 2; Figure 2), while there was no significant difference in the ROS levels in sperm cells (p>0.05, Figure 2). Therefore, 20% TEY was used in the present study.

### The effect of Equex STM paste on the quality of frozen-thawed boar sperm

When the final concentration of GLY and TEY in the Frozen Diluent II reached 5% and 20%, respectively, the TM and PM of 2% ESP group were significantly higher than those of the rest groups (p<0.05, Table 3). The VSL of 2% ESP group was slightly higher than the 0%, 1%, and 3% groups (p>0.05, Table 3), but significantly higher than the 4% groups (p<0.05, Table 3). The VCL of 2% ESP group also exhibited a slightly improvement in comparison to the 0%, 1%, and 4% groups (p>0.05, Table 3), but was significantly higher than that of the 3% group (p<0.05, Table 3). No significant differences were observed in VAP among the different groups (p>0.05, Table 3).

**Figure 3:**
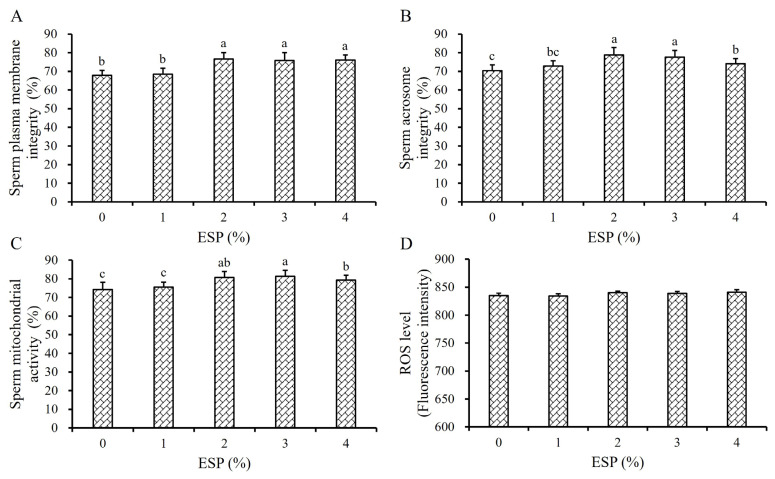
Effect of ESP on frozen-thawed boar sperm. Effect of ESP in Frozen Diluent II on the SPMI (A), SAI (B), SMA (C), and the sperm ROS level (D) respectively. The same letter or no letter indicate p>0.05. ^a–c^ Different lowercase letters indicate p<0.05. ESP, Equex STM paste.

As shown in Figure 3, the SPMI of frozen-thawed boar sperm was significantly improved when the ESP concentration in Frozen Diluent II reached 2%, 3% and 4%, compared to the 0% and 1% ESP groups (p<0.05, Figure 3A), but there were no significant differences among 2%, 3% and 4% ESP groups (p>0.05, Figure 3A). The SAI of the 2% and 3% ESP groups was significantly higher than that of the 0%, 1%, and 4% ESP groups (p<0.05, Figure 3B). The SMA of the 2% ESP group was significantly improved, compared to the 0%, 1%, and 4% ESP groups (p<0.05, Figure 3C), but there was no significant difference between the 2% and 3% ESP groups (p>0.05, Figure 3C). Additionally, the ROS levels did not exhibit significant differences across the different groups (p> 0.05, Figure 3D).

Based on these findings, the optimal ESP concentration in the Frozen Diluent II was determined to be 2% in this study.

### The effect of Mitoquinone mesylate on the quality of frozen-thawed boar sperm

When the final concentration of GLY and TEY in the Frozen Diluent II reached 5% and 20%, respectively, the TM, PM, VSL, VCL, and VAP of frozen-thawed sperm in the 250 nM and 300 nM MitoQ groups exhibited significant improvements than the 0 nM, 150 nM, and 200 nM MitoQ groups (p<0.05, Table 4), but there was no significant difference between the 250 nM and 300 nM MitoQ groups (p>0.05, Table 4).

**Figure 4:**
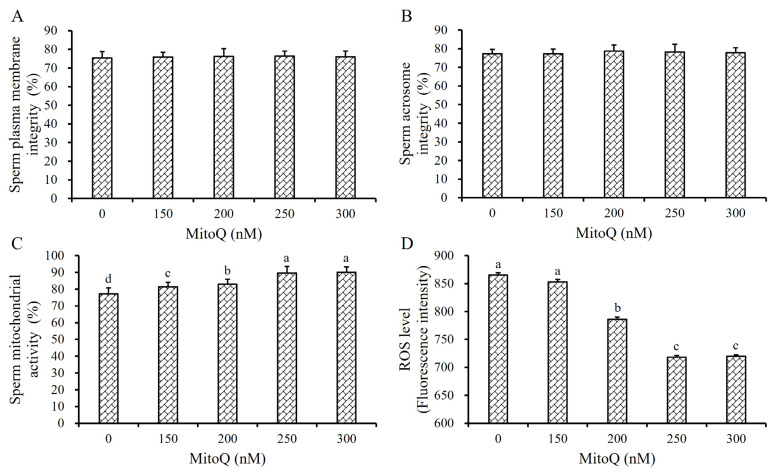
Effect of MitoQ on frozen-thawed boar sperm. Effect of MitoQ in Frozen Diluent II on the SPMI (A), SAI (B), SMA (C), and the sperm ROS level (D) respectively. The same letter or no letter indicate p>0.05. ^a–c^ Different lowercase letters indicate p<0.05. MitoQ, Mitoquinone mesylate.

As shown in Figure 4, the addition of MitoQ into the Frozen Diluent II did not significantly affect the SPMI and SAI of frozen-thawed boar sperm (p>0.05, Figures 4A, 4B), but the SMA and ROS level were significantly improved (p<0.05, Figures 4C, 4D). Specifically, when the final concentration of MitoQ in the Frozen Diluent II reached 250 nM and 300 nM, the SMA of the frozen-thawed boar sperm was significantly higher than the 0 nM, 150 nM and 200 nM MitoQ groups (p<0.05, Figure 4C). Additionally, the ROS levels in the sperm cells were significantly reduced (p<0.05, Figure 4D).

Therefore, the optimal MitoQ concentration in the Frozen Diluent II was determined to be 250 nM in this study.

### The effect of L-ergothioneine on the quality of frozen-thawed boar sperm

Compared with the 0 mM and 0.3 mM EGT groups, the TM and PM of frozen-thawed boar sperm in the 0.6 mM, 0.9 mM, and 1.2 mM EGT groups were significantly improved (p<0.05, Table 5). The VSL of the 0.6 mM and 0.9 mM EGT groups was significantly higher than the 0 mM, 0.3 mM, and 1.2 mM EGT groups (p<0.05). The VCL and VAP of the 0.6 mM group were significantly higher than the 0 mM and 0.3 mM EGT groups (p<0.05, Table 5), but there was no significant difference among the 0.6 mM, 0.9 mM, and 1.2 mM EGT groups (p>0.05, Table 5).

**Figure 5:**
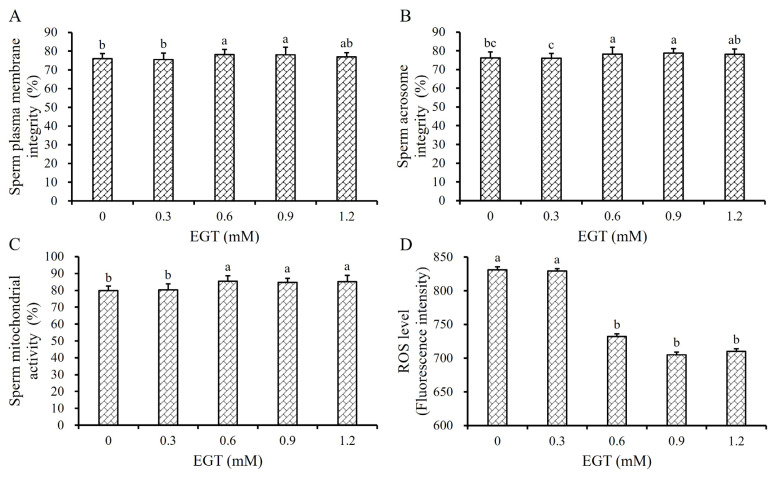
Effect of EGT on frozen-thawed boar sperm. Effect of EGT in Frozen Diluent II on the SPMI (A), SAI (B), SMA (C), and the sperm ROS level (D) respectively. The same letter indicates p>0.05. ^a–c^ Different lowercase letters indicate p<0.05. EGT, L-ergothioneine.

Like its effects on sperm motility and biokinetic parameters, the addition of different concentrations of EGT to Frozen Diluent II significantly improved the quality of sperm cells (Figure 5). When the final concentration of EGT reached 0.6 mM and 0.9 mM, the SPMI, SAI, and SMA of the frozen-thawed boar sperm were significantly higher than the 0 mM and 0.3 mM EGT groups (p<0.05), but there was no significant difference among the 0.6 mM, 0.9 mM, and 1.2 mM EGT groups (p>0.05, Tables 5A–5C). The ROS levels of 0.6 mM, 0.9 mM, and 1.2 mM EGT groups were also significantly lower than 0.9 mM and 1.2 mM EGT groups (p>0.05, Table 5D).

Therefore, the optimal EGP concentration in Frozen Diluent II was determined to be 0.6 mM in this study.

### The effect of combined cryoprotectants on the quality of frozen-thawed boar sperm

**Figure 6:**
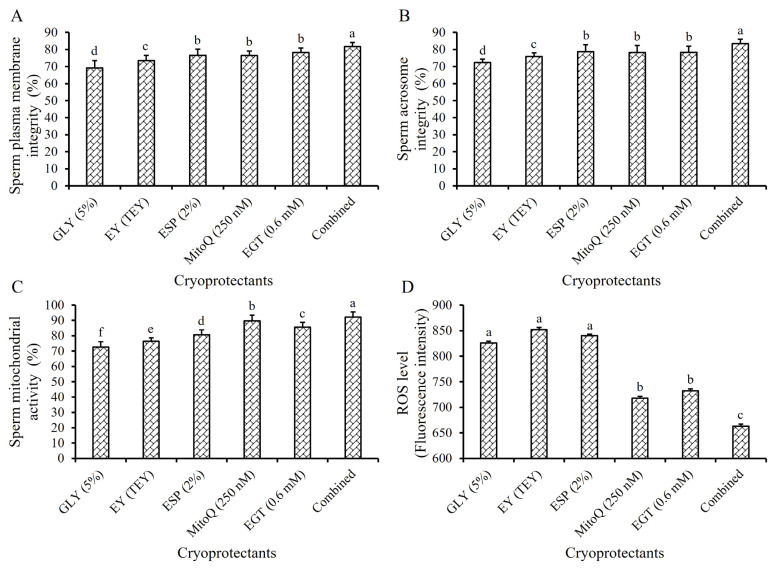
Effect of cryoprotectant alone or combined use on frozen-thawed boar sperm. Effect of cryoprotectant alone and combined use in Frozen Diluent II on the SPMI (A), SAI (B), SMA (C), and the sperm ROS level (D) respectively. The same letter indicates p>0.05. ^a–d^ Different lowercase letters indicate p<0.05. GLY, glycerol; EY, egg yolk; TEY, treated egg yolk; ESP, Equex STM past; MitoQ, Mitoquinone mesylate; EGT, L-ergothioneine.

When the five cryoprotectants were combined at their optimal concentrations in Frozen Diluent II, they exhibited a pronounced synergistic effect, the cryotolerance and the quality of frozen-thawed boar sperm were significantly improved (Table 6; Figure 6). The TM, PM, SPMI, SAI, and SMA values of sperm in the combined cryoprotectants group were significantly compared to when each cryoprotectant was used individually (p<0.05, Table 6; Figures 6A–6C). Additionally, the ROS levels in sperm cells were significantly reduced (p<0.05, Figure 6D). The VSL of the combined cryoprotectants group was not significantly improved comparing with the 0.6 mM EGT and 250 nM MitoQ groups (p>0.05, Table 6), but it was significantly higher than the 2% ESP, TEY, and 5% GLY groups (p<0.05, Table 6). Furthermore, there were no significant differences in VCL and VAP between the combined cryoprotectants group and the 250 nM MitoQ group (p>0.05, Table 6), but these parameters were significantly higher than those of the other group (p<0.05, Table 6).

### Reproductive performance of artificially inseminated sows

The FS group and FTS group with the PM exceeding 60% were utilized for artificial insemination to evaluate the impact of FTS on the reproductive performance of estrus sows. Compared with the FS group, the CR, PR, ALZ, and ALB of sows in the FTS group were not significantly affected when maintaining a consistent level of effective sperm input (p>0.05, Table 7). These data suggest that the boar semen cryopreserved using the optimized frozen diluents in this study can be effectively applied to inseminate the estrus sows in pig farm.

## DISCUSSION

During the cryopreservation of semen, sperm are subjected to various stress factors that lead to structural and functional damage. This is the primary cause of the reduced viability of frozen-thawed sperm [4]. Therefore, the key to optimizing the semen cryopreservation protocols and the composition of frozen diluent is to minimize such damage.

The process of semen freezing and thawing induces the rearrangement of water molecules both inside and outside sperm cells, then lead to the formation of ice crystals. This phenomenon has dual effects: first, the presence of large ice crystals causes irreversible mechanical damage to the structural integrity of sperm; second, the rearrangement and crystallization of water molecules create an imbalanced osmotic pressure between the intracellular and extracellular environments, resulting in partial dehydration and adverse impacts on sperm functions [14]. So, it is crucial to minimize both water molecule rearrangement and the formation of large ice crystal during freezing and thawing to mitigate potential damage to sperm. GLY is highly hydrophilic and can penetrate the cell membrane to enter sperm cells [15]. So, in semen freezing and thawing process, GLY can limit the rearrangement of water molecules, and then reduce the formation of intracellular ice crystals and the damage caused by high solute concentration, thus protecting sperm [16]. Within a certain range, the cryoprotective efficacy of GLY on sperm is positively correlated with its concentration in the frozen diluent. However, higher concentrations of GLY may also increase toxicity to sperm, thereby reducing their viability [17]. In the present study, the cryotolerance indices of post-thawed sperm in the 4% to 7% GLY group showed significant improvement compared to the 0% GLY control group, and there were no significant differences among the 5%, 6%, and 7% GLY groups (p<0.01, Table 1; Figure 1). Given that lower the concentrations of GLY in the frozen diluent result in less detrimental impact on sperm, the GLY concentration was determined to be 5% in this study. In ram semen frozen diluent, the quality of post-thawed sperm was highest when the concentration of GLY reached 6% comparing with other groups [18], and a 3% GLY concentration was recommended for buffalo bull semen frozen diluent [19]. These differences may potentially be attributed to the specific species, semen cryopreservation procedures, and the composition of the frozen diluent.

The sperm membrane is directly exposed to the liquid environment during the semen freezing process, making it highly susceptible to cold shock [20]. The liquid crystalline phase of the sperm plasma membrane is crucial for its biological function [21]. As the temperature of semen decreases from 25°C to 5°C, the sperm plasma membrane gradually transitions from the liquid crystalline phase to the gel phase, leading to the tight arrangement of phospholipid molecules in an ordered gel state and the loss of normal biological function. This is a significant factor contributing to the decline in post-thawed sperm quality [22]. The plasma membrane of boar sperm, which is rich in polyunsaturated fatty acids, is particularly sensitive to cold shock [23]. EY contains high levels of lecithin, low-density lipoprotein, and other unverified functional components, which can protect the sperm membrane against cold shock and significantly improve the post-thawed sperm viability [24]. To date, despite the widespread use of EY as an effective cryoprotectant, the exact mechanism by which it protects against cold shock remains poorly understood due to its highly complex composition [25]. Existing studies have indicated that EY may act at the sperm cell membrane level by inducing resistance against cold shock and protecting against freeze damage [26]. The active components in EY are primarily present as large spherical particles [27]. Therefore, we hypothesized that the protective effect of EY on the sperm plasma membrane could be enhanced if the substances within the EY granules were adequately released and came into closer contact with the sperm cell membrane. To achieve this, we employed two methods: one involved using a high-pressure homogenizer to disrupt the EY particles [28], and the other entailed adding the surfactant ESP to fully emulsify and evenly distribute the fat-soluble substances in EY into the frozen diluent [29]. Our results confirmed our hypothesis. Compared to the UTEY group, the SPMI, SAI, and SMA of post-thawed in the TEY group were significantly improved (p<0.05, Figures 2A–2C), indicating that TEY provided superior protection against freezing-induced damage to the sperm plasma membrane. Additionally, the significant improvement of biokinetic parameters VSL, VCL, and VAP of post-thawed sperm in the TEY group (p<0.05, Table 2) further supported this conclusion. Similarly, as the concentration of ESP in the diluent increased, its protective effect on sperm progressively enhanced until it reached a concentration of 2%. Beyond this point, further increases in ESP concentration led to a decline in its protective effect (Table 3; Figure 3). This phenomenon may be attributed to surfactant properties of ESP, excessively high concentrations of ESP in the diluent could disrupt the stability of the lipid bilayer of the sperm plasma membrane, thereby adversely affecting sperm viability [29]. When 20% TEY and 2% ESP were simultaneously added to the frozen diluent, the quality of post-thawed boar sperm showed even greater improvement (p<0.05, Table 3; Figure 3). However, there was no significant change in ROS levels in the TEY and ESP groups (p>0.05, Figures 2D, 3D), suggesting that their protective effects were not mediated by reducing peroxide reactions.

Oxidative stress is another critical factor contributing to damage in sperm structure [30]. During the freezing and thawing of semen, an abundance of ROS and free radicals are generated, leading to the oxidation of phospholipids, proteins, and polysaccharides within the sperm plasma membrane [31]. This results in decreased fluidity, stability, and semi-permeability of the plasma membrane, disrupting normal biological functions [32]. Therefore, adding antioxidant to the frozen diluent can effectively mitigate the oxidative stress suffered by sperm during freezing and significantly improve the quality of post-thawed sperm [33]. MitoQ, a mitochondria-targeted antioxidant, has been shown to substantially reduce mitochondrial ROS levels and minimize oxidative damage [34]. MitoQ has been successfully utilized as an effective cryoprotectant for the cryopreservation of semen from different species, including goat [35], ram [36], rooster [37], and buck [38] semen. In this study, the motility and biokinetic parameters of the 250 nM MitoQ group were significantly improved (p<0.05, Table 4), indicating its protective effect on the cryopreservation of boar sperm. However, the optimal dosage varies considerably across different species [35–38]. Furthermore, the SPMI and SAI of did not exist significant difference among different groups (p>0.05, Figures 4A, 4B), but the SMA of the 250 nM MitoQ groups was significantly improved while the ROS level in sperm cell of 250 nM MitoQ groups was significantly reduced (p<0.05, Figures 4C, 4D). These results suggested that MitoQ primarily exerts a protective effect on the structure and function of mitochondria, without apparent protection on the plasma membrane of sperm. To protect the sperm plasma membrane against oxidative stress-induced damage, we evaluated the effect of another antioxidant, EGT, on cryopreserved sperm. EGT is a highly efficient antioxidant, and widely distributed in animal tissues and organs [39], and had a significant protective effect on the cryopreservation of rooster and ram sperm [40]. The result showed that the quality of post-thawed sperm was significantly improved when 0.6 mM EGT were added (p<0.05, Table 5; Figure 5). But the effect of EGT on frozen-thawed sperm differed from that of MitoQ, as it also enhanced the integrity of the plasma and acrosome membrane, suggesting its role as a non-targeted antioxidant (p<0.05, Figures 5A, 5B).

## CONCLUSION

In this study, adding 5% GLY, 20% TEY, 2% ESP, 250 nM MitoQ, and 0.6 mM EGT to a Glucose-Sodium citrate-Tris based frozen diluent separately, the quality of frozen-thawed boar sperm was significantly improved. When these five cryoprotectants were combined, they showed a marked synergistic effect, the quality of frozen-thawed boar sperm existed significant improvement than they were added separately. Using the optimized cryopreservation system to cryoprotect pig sperm and artificially inseminate the estrus sow, the reproductive performance of the sows was not significantly different from that of the sows inseminated with FS, indicating that it can be applied to the artificial insemination of sows in pig farms. Our research provides a preferred technical solution for the cryopreservation of excellent boar’s semen, as well as the gene exchange and interregional collaborative breeding.

## Figures and Tables

**Table 1:** Effect of GLY on the motility and biokinetic parameters of frozen-thawed boar sperm

GLY (%)	TM (%)	PM (%)	VSL (μm/s)	VCL (μm/s)	VAP (μm/s)
0 (Control)	2.25±0.89^B^	1.03±0.57^B^	20.43±2.09^B^	32.29±2.64^B^	25.64±1.87^B^
4	53.63±1.29^Ab^	42.27±1.00^Ac^	38.68±1.87^Ab^	51.02±1.90^Ab^	40.32±2.06^Ab^
5	62.25±1.36^Aa^	58.73±1.61^Aa^	47.62±2.36^Aa^	63.56±2.19^Aa^	53.17±1.70^Aa^
6	61.85±1.10^Aa^	57.35±0.95^Aa^	44.76±2.58^Aa^	63.11±1.32^Aa^	52.94±1.93^Aa^
7	60.92±1.21^Aa^	50.87±1.33^Ab^	46.01±1.65^Aa^	62.61±1.66^Aa^	51.86±0.96^Aa^

Different superscript lowercase letters (a–c) in the same column differ at p<0.05 and different superscript capital letters (A,B) in the same column differ at p<0.01.

GLY, glycerol; TM, total motility; PM, progressive motility; VSL, straight-line velocity; VCL, curvilinear velocity; VAP, average path velocity.

**Table 2:** Effect of the EY on the motility and biokinetic parameters of frozen-thawed boar sperm

EY (20%)	TM (%)	PM (%)	VSL (μm/s)	VCL (μm/s)	VAP (μm/s)
UTEY	62.32±0.98^a^	58.16±1.22^a^	48.84±1.90^b^	62.88±1.89^b^	54.06±2.34^b^
TEY	64.89±1.10^a^	62.08±1.53^a^	59.37±2.64^a^	76.21±2.51^a^	65.11±2.08^a^

Different superscript letters (a,b) in the same column differ at p<0.05.

EY, egg yolk; EG, experimental group; TM, total motility; PM, progressive motility; VSL, straight-line velocity; VCL, curvilinear velocity; VAP, average path velocity; UTEY, untreated egg yolk; TEY, treated egg yolk.

**Table 3:** Effect of ESP on the motility and biokinetic parameters of frozen-thawed boar sperm

ESP (%)	TM (%)	PM (%)	VSL (μm/s)	VCL (μm/s)	VAP (μm/s)
0	63.36±1.16^b^	59.23±1.56^c^	60.31±1.20^a^	77.33±1.56^ab^	68.11±1.92^a^
1	63.95±1.35^b^	59.69±2.10^c^	59.48±1.44^ab^	77.92±2.30^ab^	68.54±2.30^a^
2	70.12±1.60^a^	69.38±1.72^a^	61.23±1.15^a^	79.89±2.63^a^	69.02±1.05^a^
3	69.63±2.06^a^	65.14±0.96^ab^	61.19±2.18^a^	75.06±1.27^b^	67.98±0.98^a^
4	66.02±1.28^ab^	63.52±1.17^bc^	57.66±1.03^b^	77.53±1.18^ab^	68.62±1.35^a^

Different superscript letters (a–c) in the same column differ at p<0.05.

ESP, Equex STM paste; TM, total motility; PM, progressive motility; VSL, straight-line velocity; VCL, curvilinear velocity; VAP, average path velocity.

**Table 4:** Effect of MitoQ on the motility and biokinetic parameters of frozen-thawed boar sperm

MitoQ (nM)	TM (%)	PM (%)	VSL (μm/s)	VCL (μm/s)	VAP (μm/s)
0	64.66±1.30^b^	60.87±0.96^b^	58.26±2.18^c^	75.58±2.08^c^	69.08±2.47^c^
150	63.93±2.01^b^	60.12±1.67^b^	62.32±1.27^b^	79.13±1.46^bc^	72.43±2.62^b^
200	65.01±1.72^b^	61.83±2.14^b^	62.14±0.80^b^	80.60±1.97^b^	72.55±1.17^b^
250	71.96±1.54^a^	69.36±1.52^a^	67.32±2.06^a^	84.84±2.05^a^	76.91±1.40^a^
300	71.27±1.10^a^	69.14±0.86^a^	67.42±1.55^a^	84.02±1.18^a^	76.06±2.51^a^

Different superscript letters (a–c) in the same column differ at p<0.05.

MitoQ, Mitoquinone mesylate; TM, total motility; PM, progressive motility; VSL, straight-line velocity; VCL, curvilinear velocity; VAP, average path velocity.

**Table 5:** Effect of EGT on the motility and biokinetic parameters of frozen-thawed boar sperm

EGT (mM)	TM (%)	PM (%)	VSL (μm/s)	VCL (μm/s)	VAP (μm/s)
0	63.99±2.09^b^	59.15±1.52^b^	60.30±1.40^b^	74.32±1.65^b^	66.76±2.34^b^
0.3	64.08±1.30^b^	60.87±2.14^b^	61.75±2.76^b^	75.99±2.08^b^	66.89±1.80^b^
0.6	70.55±1.55^a^	68.15±1.35^a^	66.95±1.54^a^	79.13±2.14^a^	71.75±1.42^a^
0.9	70.22±0.94^a^	68.20±0.86^a^	66.11±1.97^a^	78.46±1.35^ab^	71.07±0.98^a^
1.2	69.96±1.35^a^	67.82±1.65^a^	61.01±2.19^b^	76.90±1.57^ab^	69.29±1.94^ab^

Different superscript letters (a,b) in the same column differ at p<0.05.

EGT, L-ergothioneine; TM, total motility; PM, progressive motility; VSL, straight-line velocity; VCL, curvilinear velocity; VAP, average path velocity.

**Table 6:** Effect of combined cryoprotectants on the motility and biokinetic parameters of frozen-thawed boar sperm

Cryoprotectants	TM (%)	PM (%)	VSL (μm/s)	VCL (μm/s)	VAP (μm/s)
GLY (5%)	62.25±1.36^c^	58.73±1.61^c^	47.62±2.36^c^	63.56±2.19^c^	53.17±1.70^d^
EY (TEY)	64.89±1.10^c^	62.08±1.53^c^	59.37±2.64^b^	76.21±2.51^b^	65.11±2.08^c^
ESP (2%)	70.12±1.60^b^	69.38±1.72^b^	61.23±1.15^b^	79.89±2.63^b^	69.02±1.05^bc^
MitoQ (250 nM)	71.96±1.54^b^	69.36±1.52^b^	67.32±2.06^ab^	84.84±2.05^a^	76.91±1.40^a^
EGT (0.6 mM)	70.55±1.55^b^	68.15±1.35^b^	66.95±1.54^ab^	79.13±2.14^b^	71.75±1.42^b^
Combined	76.92±1.43^a^	75.88±1.10^a^	68.17±2.11^a^	85.36±1.87^a^	78.16±2.01^a^

Different superscript letters (a–d) in the same column differ at p<0.05.

TM, total motility; PM, progressive motility; VSL, straight-line velocity; VCL, curvilinear velocity; VAP, average path velocity; GLY, glycerol; EY, egg yolk; TEY, treated egg yolk; ESP, Equex STM past; MitoQ, Mitoquinone mesylate; EGT, L-ergothioneine.

**Table 7:** Reproductive outcomes of artificially inseminated sows using cryopreserved boar sperm

Group	NIS (n)	NFS (n)	NPS (n)	CR (%)	PR (%)	ALZ	ALB
FS	50	46	46	92.0	92.0	13.5±1.9	13.2±1.1
FTS	50	45	44	90.0	88.0	12.9±2.1	12.6±1.3

The absence of superscript letters in the same column indicates p>0.05.

NIS, number of inseminated sows; NFS, number of fertilized sows; NPS, number of parturient sows; CR, conception rate; PR, parturition rate; ALZ, average litter size; ALB, average number of live births; FS, fresh semen; FTS, frozen-thawed semen.
